# The circuitry of the tumor microenvironment in adult and pediatric Hodgkin lymphoma: cellular composition, cytokine profile, EBV, and exosomes

**DOI:** 10.1002/cnr2.1311

**Published:** 2020-10-26

**Authors:** Poonam Nagpal, Dante B. Descalzi‐Montoya, Niraj Lodhi

**Affiliations:** ^1^ College of Natural, Applied, and Health Sciences Kean University Union New Jersey USA; ^2^ Center for Discovery and Innovation, The John Theurer Cancer Center Hackensack‐Meridian Health Nutley New Jersey USA; ^3^ Department of Immunotherapeutics and Biotechnology Texas Tech University Health Science Center Abilene Texas USA

**Keywords:** EBV, Hodgkin lymphoma, pediatric, tumor microenvironment

## Abstract

**Background:** Classical Hodgkin lymphoma (cHL) is a unique lymphoid malignancy with a tumor microenvironment (TME) consisting of a small number of neoplastic—Hodgkin and Reed‐Sternberg (H‐RS) cells (<1%), surrounded by a large number of nonneoplastic infiltrating immune cells (>90%). The TME of cHL critically depends on immune cells to support tumor growth as H‐RS cells cannot survive and proliferate in isolation.

**Recent Findings:** Programmed cell death protein 1 (PD‐1) ligand expressed on H‐RS cells inhibits the clearance of tumor by causing T‐cell exhaustion. Nivolumab and pembrolizumab, PD‐1 inhibitors, have been proven to be effective in treating adult and pediatric patients with R/R cHL. Tumor‐associated macrophages (TAMs) are a central component of TME and are known to cause poor prognosis in adult HL. However, the prognostic impact of CD68+ TAMs in pediatric HL remains ambiguous. EBV modulates the tumor milieu of HL and plays a strategic role in immune escape by enrichment of the TME with *T*_*reg*_ cells and associated immunosuppressive cytokines in adult HL. In contrast, EBV+ pediatric patients have increased infiltration of CD8^+^ T‐cells and show a better therapeutic response suggesting viral‐related TME is distinct in childhood HL. The role of CASP3 in apoptosis of H‐RS cells and its correlation with response prediction in adult and pediatric HL suggest it may serve as a potential biomarker. In cHL, CD30, EBV, and NF‐κB signaling employ exosomes for cell–cell communication that triggers the migration capacity of fibroblasts, stimulate to produce proinflammatory cytokines, and help to create a tumor‐supportive microenvironment.

**Conclusion:** The cHL microenvironment is distinct in adult and pediatric HL. Future studies are required to understand the role of interplay between H‐RS cells and EBV‐associated microenvironment and their clinical outcome. They may present novel therapeutic targets for the development of antilymphoma therapy.

## 1 INTRODUCTION

Classical Hodgkin lymphoma (cHL) is a lymphoid malignancy with malignant cells, Hodgkin and Reed‐Sternberg (H‐RS), constituting only a small percentage of a tumor, and these cells are surrounded by a large number of nonneoplastic infiltrating cells (>90%) including lymphocytes, neutrophils, eosinophils, mast cells, macrophages, and fibroblasts. The clinicopathological features include B symptoms resulting from deregulated cytokines and chemokines produced mainly by H‐RS cells and, to some extent by infiltrating cells, leading to an immunosuppressive tumor microenvironment (TME). Despite the high cure rate with first‐line therapy (ABVD), 5% to 10% of patients are refractory and 10% to 30% of patients relapse. High‐dose chemotherapy followed by autologous stem cell transplant is a standard of care for relapsed/refractory (R/R) HL patients which leads to a cure in about 50% of patients. Immunotherapy with checkpoint inhibitors has revolutionized the treatment of several cancers. Program cell death protein‐1 (PD‐1) is currently the most widely studied inhibitory checkpoint molecule. HRS cells express PD‐1 ligands, which bind to PD‐1 receptors expressed on peritumoral T‐cells, thereby contributing evasion of immune detection by inhibiting T‐cell receptor signaling. Nivolumab and pembrolizumab, monoclonal antibodies to PD‐1, have been proven to be effective in treating patients with R/R cHL. Treatment with nivolumab in phase I and II trials of R/R cHL patients showed an overall response rate (ORR) of 87% and 66%, respectively.^1^, ^2^ Likewise, pembrolizumab therapy in R/R CHL patients was associated with high response rates and an acceptable safety profile.^3^, ^4^ In phase II study of R/R cHL patients who had failed ASCT, pembrolizumab was associated with PFS and OS of 82% and 100%, respectively, at 18‐months.^5^ In pediatric HL patients also, nivolumab and pembrolizumab have proven to be safe and well‐tolerated. In the phase I trial of R/R pediatric HL patients, nivolumab and pembrolizumab showed an objective response rate of 30% and 60%, respectively.^6^, ^7^ Further investigations of PD‐1 inhibitors combined with other therapeutic agents may offer an effective cure in pediatric HL patients.

Current strategies in first‐line treatment aim to improve the outcome and prevent treatment‐related toxicity, including reproductive infertility, cardiopulmonary toxicity, and secondary malignancy. Pediatric patients may be overtreated by using unnecessary aggressive regimens. Pediatric HL is different from adult HL in terms of the relative incidence of specific histological subtypes and cellular composition of the TME thereby causing a distinct immune profile against H‐RS cells.^8^ There is considerable variability in the TME across various histological subtypes. Therefore, it is imperative to understand the biology of the TME in adult and pediatric HL to tailor the treatment accordingly and to prevent long‐term side effects in children.^9^ Pediatric HL research has been hampered by the lack of any cell line model and limited availability of patient samples. Furthermore, adult HL studies may not be completely applicable to pediatric HL patients. Previous research studies have focused on differences between adult and pediatric HL with respect to histological subtypes, TAMs, EBV, and various biomarkers. However, the detailed descriptions of overall TME components differences between adult and pediatric HL are rare. Here, the current review discusses the components that constitute and modulate distinctly the tumor microenvironment in adult and pediatric HL including: (a) cellular composition, (b) cytokine profile, (c) Epstein–Barr virus (EBV), and (d) exosomes.

## 2 TME MODULATORS

### 2.1 Cellular composition

**FIGURE 1:**
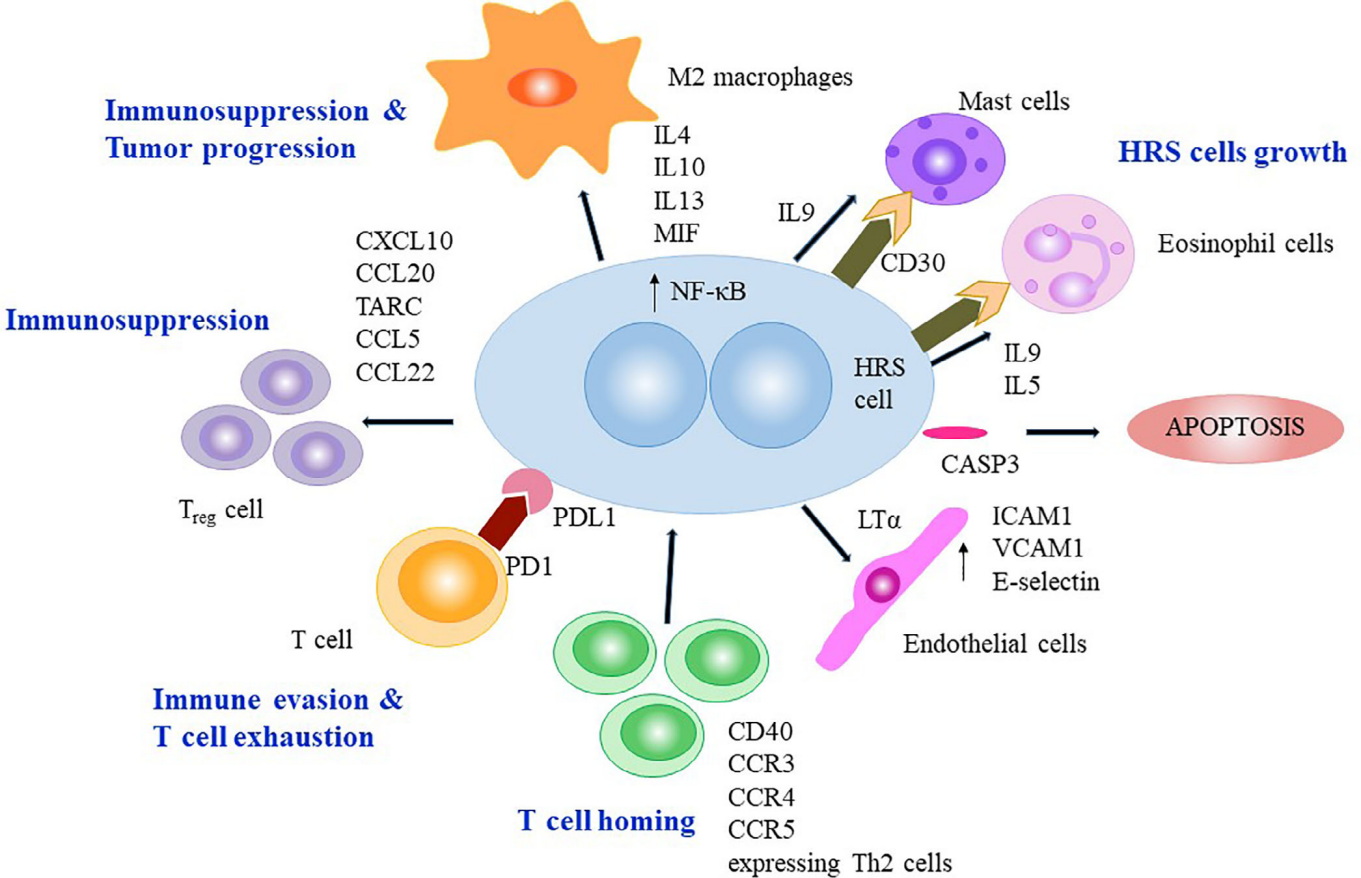
Schematic representation of tumor microenvironment in cHL depicting how H‐RS cells are involved in recruitment, immune evasion, and reprograming of various other infiltrating cells. H‐RS cells secrete CXCL10, CCL20, TARC (CCL17), CCL5/RANTES, and CCL22/MDC that attract *T*_*reg*_ cells. H‐RS cells secrete LTα to attract endothelial cells which in turn assist in T‐cell infiltration. On the other side, Th2 lymphocytes cells also get recruited by expressing CD40, CCR3, CCR4, CCR5 receptors to the corresponding ligand produced by H‐RS cells. M2 macrophages are attracted to the tumor microenvironment by H‐RS cells through the secretion of cytokines IL4, IL10, IL13, and MIF thereby promote tumor growth. IL5 and IL9 promote growth and differentiation of eosinophil and mast cells in TME. IL5 levels correlate with tissue eosinophilia commonly seen in cHL. H‐RS cells play a role in immune evasion by expressing PDL1 that binds to PD1 on T‐cells resulting in inhibition of antitumor immunity. The expression of CASP3+ on H‐RS cells leads to apoptosis and correlates with better survival

In the tumor milieu of HL, infiltrating T‐cells rosette around H‐RS cells but are ineffective in eliminating malignant H‐RS cells. The cellular composition typically includes T‐helper (Th)2 and regulatory T‐cells (*T*_*reg*_), and low Th1 cells, CD8+ cytotoxic T cells (CTL), and natural killer (NK) cells, thereby causing a shift toward an immunosuppressive environment and preventing cytotoxic antitumor immune responses.^10^, ^11^ However, a recent study showed the presence of Th1 type, CXCR3+ cells in cHL patients, and increased production of tumor necrosis factor‐alpha (TNF‐α) from reactive T‐cells, yet the level of IL‐21, a Th2 cytokine also increased.^12^ Thus, it appears that a Th2 cell–dominated microenvironment favors the survival and progression of tumor. H‐RS cells secrete T‐cell homing molecules, adhesion molecules, and endothelial activation proteins to induce proliferation and blood vessel formation. There is compelling evidence to show that T‐cells crosstalk with neoplastic cells within the TME.^13^, ^14^ In addition, H‐RS cells can secrete molecules that negatively impact CTL via Fas–FasL interaction and Galectin‐1.^15^, ^16^ HL‐associated fibroblasts (HL‐AF) exhibit an inflammatory phenotype and upregulated expression of alpha‐smooth muscle actin (αSMA), which is involved in fibroblast contractile activity.^17^ HL‐AFs release growth factors and proinflammatory cytokines such as IL‐1α, IL‐6, and TNF‐α into the TME to support tumor growth and maintenance (Figure 1).^18^ Tumor‐associated macrophages (TAMs) in the TME are largely M2‐polarized and are activated by Th2 anti‐inflammatory cytokines such as IL‐4, IL‐10, and IL‐13 and macrophage migration inhibitory factor (MIF).^19^ TAMs have been shown to promote an antiinflammatory response, proliferation, angiogenesis, matrix remodeling, tumor growth, and metastasis. Steidl et al,^20^ first assessed the prognostic impact of TAMs and showed that a macrophage gene expression signature is associated with inferior outcome in cHL patients. This analysis was further validated in a patient cohort with increased CD68 expression, a characteristic marker of TAMs. Subsequently, many studies have confirmed the association of TAMs with poor prognosis of adult HL patients (Table 1).^21^, ^22^, ^23^, ^24^ A couple of studies investigated the impact of TAMs on prognosis of pediatric patients. In contrast to adult HL patients, TAMs failed to predict disease outcome in pediatric patients.^8^, ^25^, ^26^ However, Barros et al showed that high numbers of CD163+ macrophages were associated with worse progression‐free survival in EBV– cases but not in EBV+ cases (discussed below).^27^ Possibly, in EBV‐associated TME of pediatric HL, macrophages are M1‐polarized and therefore may mediate effective immune surveillance. TAMs may have hormetic rather than linear relationship to outcome in HL. Recently, it is shown that a small number of TAMs may have a moderate growth promoting effect on cHL, while with increasing numbers, macrophages display an inhibitory effect and only become supportive of tumor growth above a certain threshold.^28^ Further investigation may provide insight into relationship of TAMs with treatment outcome in adult Hodgkin lymphoma and other tumors.

**TABLE 1:** Comparison of histological subtypes and tumor microenvironment modulators between adults and pediatric HL

Factors	Adult	Pediatric	Reference
Relative incidence			
Nodular sclerosis	75%–80%	40%–45%	93
Mixed cellularity	15%–20%	30%–45%	93
TME modulators			
Cellular composition			
TAM	High levels of CD68+ TAMs indicate poor prognosis	CD68+ failed to predict disease outcome. Higher number of CD163+ TAMs associated with poor survival in EBV– patients	21, 23, and 25, 26, 27
Cytokines			
TARC	High levels indicate Poor prognosis	Diagnostic and disease response marker	39 and 51
IL6	“B” symptoms, poor prognosis, interim prognostic marker	IL6 expression on background cells is predictor of treatment failure	44, 52, and 94
IL10	B symptoms, adverse therapeutic response	B symptoms, adverse therapeutic response, influences composition of TME	33, 34, 44, 50, and 54
VEGF	Prognostic marker, increased microvessel density, tumor progression, association with EBV	Prognostic marker	56, 57, 58
EBV	EBV+ cHL associated with adverse outcome	EBV− pediatric cHL display poor outcome	20, 27, and 28
Exosomes	Interfere with host immune response, prognostic significance	ND	88 and 89

### 2.2 Cytokines

Cytokines, produced by H‐RS cells, contribute to HL pathogenesis, in an autocrine and paracrine manner, and help to recruit and sustain reactive T‐cells. These cytokines are inducers of HL milieu—IL‐4,^29^ IL‐5^30^; growth factors—IL‐6, IL‐9,^31^ IL‐13^32^; and anti‐inflammatory—IL‐10,^33^, ^34^ tumor growth factor β (TGF‐ β).^35^ Additionally, H‐RS cells produce Th2 and *T*_*reg*_ chemoattractants such as TARC (CCL17),^34^, ^36^ CCL5/ RANTES,^37^ and macrophage‐derived chemokine (MDC/CCL22).^38^ TARC levels increase in the sera of cHL patients and reflect tumor status since elevated levels decrease during treatment in most clinical responders. In a recent study of combined adult and pediatric HL, early reduction in TARC1 levels in combination with interim PET scan was predicted as the success of response (Table 1).^39^ Tumor‐infiltrating T‐cells also express T‐cell homing receptors such as CCR3, CXCR4, CCR5, and CCR7, corresponding to ligands expressed by H‐RS cells.^13^ H‐RS cells exploit the immune evasion mechanism to survive and prevent antitumor immunity. Programmed cell death protein 1 (PD‐1) ligand expressed on H‐RS cells blocks T‐cell effector functions by binding to its receptor on T‐cells (T‐cell exhaustion), thus impeding the clearance of the tumor (Figure 1).^35^, ^40^ Receptors and ligands belonging to the TNF superfamily, for example, TNF‐α, lymphotoxin‐alpha (LTα), CD30, and CD40, also play an important role in the pathogenesis of cHL. TNF‐α confers poor prognosis and is associated with advanced stage, “B” symptoms, response failure, and decreased survival.^41^ LTα gene expression has been found in cHL lymph nodes and H‐RS cells.^42^ LTα activates endothelial cells to upregulate the expression of adhesion molecules including hyaluronan, ICAM1, VCAM‐1, and E‐selectin which mediate T‐cell recruitment.^14^ CD30 is expressed on H‐RS cells in nearly 100% cHL cases, and high expression of CD30 has been associated with poor prognosis.^43^ Furthermore, prior to diagnosis, elevated levels of soluble‐CD30 (sCD30), in conjunction with IL‐6 and IL‐10, have been associated with a significantly high risk of cHL and promote lymphomagenesis.^44^ Indeed, CD40L is expressed on T‐cell rosettes and activation of its receptor, and CD40 results in increased NF‐κB signaling and colony formation of H‐RS cells, thereby providing survival signals.^45^, ^46^ TGF‐β has been detected predominantly in nodular sclerosis (NS) HL and has potent anti‐inflammatory properties.^35^, ^47^ Caspase3 (CASP3) is a marker of cell death by apoptosis, and the fact that high levels of CASP3^+^ H‐RS cells correlate with a better outcome in adult^48^ and pediatric^49^, ^50^ HL underscores its role in apoptosis of H‐RS cells. Thus, CASP3 can serve as a potential biomarker for response prediction of HL. Moreover, CASP3 gene expression is associated with other TME‐associated genes such as GrB and lysozyme (LYZ). Cytokine profiling in pediatric HL revealed that CD30, IL‐10, IL‐6, ICAM‐1, VEGF, and TARC are independent prognostic markers (Table 1).^9^, ^51^, ^52^ High serum CD30 levels correlated with advanced stage, B symptoms, tumor bulkiness, and treatment failure^53^ suggesting sCD30 plays a crucial role by mediating cross‐talk in the HL tumor microenvironment. High levels of serum IL‐10 and polymorphisms are associated with B symptoms and adverse therapeutic response in both adults and childhood HL indicating its direct role in cHL pathogenesis.^54^ Additionally, polymorphism in IL‐10 affects host genetic susceptibility and influences the composition of the TME in pediatric HL.^55^ IL‐6 expression in the background cells is an independent poor predictor of response in pediatric HL.^52^ VEGF is a well‐known angiogenic marker, and high circulating VEGF levels have been associated with high microvascular density and treatment failure in both adult and pediatric HL.^56^, ^57^, ^58^ Overall, pleiotropic cytokines play one of the most important roles in immune evasion in HL.

### 2.3 EBV

**FIGURE 2:**
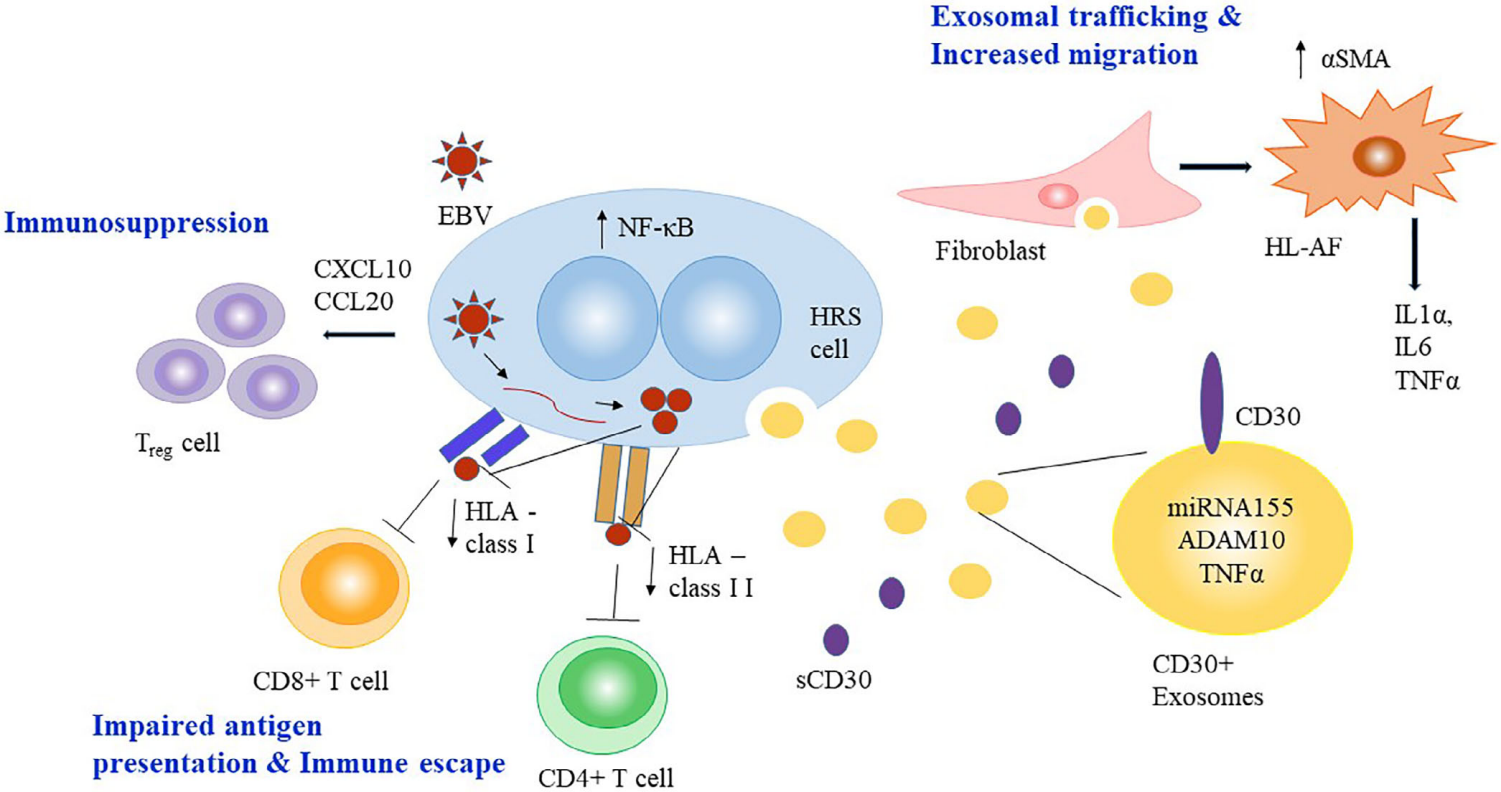
Schematic illustration showing the role of EBV and exosomes in cell–cell communication and in turn leading to HL tumor growth and dissemination. CD30+ exosomes trafficking to fibroblast support HL tumor dissemination. ADAM10 enhances CD30 shedding that interferes with host immune surveillance. EBV modulates the TME resulting in an impaired immune response

EBV is present in tumor cells in about 40% of cHL patients in developed countries and plays a crucial role in cHL pathogenesis. EBV positivity is frequently observed in childhood (<10 years) and in older adults (>60 years) HL patients and is highest in mixed cellularity (~75%) type.^59^ H‐RS cells positive for EBV reveal type II latency phenotype, expressing a limited number of latency genes, latent membrane protein (LMP)1, LMP2A, EBNA1, and EBER‐1/2. LMP1, an oncogene product, mimics CD40 that stimulates NF‐κB pathway activation,^60^ and LMP2A can substitute the function of BCR,^61^ thereby immortalizing B‐cells that are otherwise destined to undergo apoptosis. The presentation of peptides from these EBV proteins is mediated through human leukocyte antigens (HLA) classes I and/or II. Both CTL and CD4+ T‐cells that recognize HLA class I and class II, respectively, are involved in an antitumor immune response against EBV.^62^ Of note, HLA class I region polymorphism is known to have consistent susceptibility effects on sporadic and familial EBV‐positive childhood and adult HL across different geographical locations.^63^, ^64^, ^65^, ^66^, ^67^, ^68^, ^69^ The classic case study of familial HL demonstrated that clustering of EBV and certain identical HLA genotypes in pediatric patients with HL can occur in a single‐family^66^ in which three siblings were diagnosed with EBV‐positive HL had different HLA class I phenotype from their two unaffected siblings. These studies suggest that the antigenic presentation of EBV‐derived peptides is involved in the development of HL (Figure 2). In adult HL patients, EBV+ H‐RS cells have been shown to stimulate chemokines such as CXCL10 and CCL20 that attract *T*_*reg*_ cells in the TME.^70^, ^71^ Likely, these *T*_*reg*_ cells (FOXP3+ cells) further inhibit the migration and differentiation of GrB+ cells. EBV+ tumors exhibit enhanced production of IL‐10. Additionally, molecular gene profiling of EBV+ tumors has revealed the overexpression of IFN‐γ, CXCL9, CXCL10, and CXCL11/ITAC that may drive Th1 reaction in the cHL microenvironment.^72^ Interestingly, EBV+ cases with Th1 reaction did not show a better prognosis. In contrast, EBV+ pediatric patients exhibited a cytotoxic profile characterized by increased infiltration of CD8^+^ T cells and showed a better therapeutic response.^8^, ^73^, ^74^, ^75^ However, a subset of EBV+ patients showed worse outcome with a high number of FOXP3+ cells, suggesting EBV modulates the immune escape strategies by enrichment of the TME with *T*_*reg*_ cells and associated, immunosuppressive cytokines.^76^ This outcome could be attributed to an insufficient intratumoral immune response against EBV in adult HL that fails to remove tumor cells. This holds true for other types of EBV+ lymphomas also, where it is shown that subsets of circulating T‐cells are immune exhausted and unable to produce principal cytokines‐mediating innate immunity against viral infection.^77^, ^78^, ^79^ Therefore, regardless of a Th1 or Th2 dominant TME, the final impact on T‐cells subsets is an overall immunological suppression exerted by *T*_*reg*_ cells which inhibit the induction of tumor immunity. Primary immunodeficiencies due to alterations in interleukin‐2‐inducible T‐cell kinase (*ITK*) gene,^80^, ^81^ CD70, and its receptor, CD27 has been well documented with EBV‐associated lymphoma.^82^ An interesting study, using whole‐genome sequencing, revealed that EBV‐associated pediatric and adolescent HL cases had autosomal‐recessive CD70 deficiency.^83^ CD70 binds to CD27 which is expressed on human naive and some memory T‐cell subsets, germinal center and memory B‐cells, plasma cells, and a subset of NK cells.^84^ Mutations in CD70 blocked either its expression or interaction with CD27, and patients were presented with reduced EBV‐specific effector memory CD8+ T‐cells suggesting its role in T‐and B‐cell‐mediated immunity, especially for protection against EBV and humoral immunity. In general, EBV+ HL patients have demonstrated mixed therapeutic responses including both favorable and unfavorable prognostic response. Nevertheless, EBV plays a key role in modulating the TME and that may influence immune response against EBV infection and H‐RS cells.

### 2.4 Exosomes

Exosomes are the smallest type of extracellular vesicles (EVs) (30‐150 nm) that are released from cells into the adjacent microenvironment after the peripheral membrane of an endosome fuses with the plasma membrane. Exosomes are carriers of diverse exosomal cargoes such as proteins, DNA, mRNA, and microRNAs (miRNA) that facilitate cell–cell communication in tumor development and progression.^85^ Exosomes in B‐cell lymphomas participate in both antitumor immune responses as well as evasion from immunosurveillance and immunosuppression. In addition, they are recognized as serum biomarkers to detect tumor characteristics and monitor chemotherapy efficacy. The underlying mechanism involving exosomes in tumor growth has been recently explored. Mesenchymal cells secrete CCR2 and recruit TAMs in the TME that result in tumor growth facilitated by exosomes.^86^ HL cells–derived CD30+ EVs are internalized by fibroblasts resulting in increased migration accompanied by the induced release of cytokines that support HL tumor progression (Figure 2).^18^ Considering well‐documented studies that CD30 is universally expressed on H‐RS, its involvement in exosome trafficking is not surprising. H‐RS cells are thought to form tubulin‐based protrusions that govern the polarization of CD30+ extracellular vesicles to the target cells such as eosinophils and mast cells, which become effective upon induction of CD30 signaling.^87^ CD30 shedding is also enhanced by metalloproteinase‐ADAM10, after it is released from exosome‐like vesicles by HL cells and lymph node mesenchymal stromal cells and interferes with host immune responses (Table 1).^88^ In cHL tissues, tumor‐derived exosomes showed elevated signals for miR21‐5p, miR127‐3p, let7a‐5p, miR24‐3p, and miR155‐5p. Notably, miR155 levels were decreased significantly after chemotherapy.^89^ EBV is recently emerging as a key player that could hijack the exosome pathway to release Fas ligand and induce apoptosis of T‐cells and B‐cells resulting in T‐cell depression and immune evasion.^90^ The mechanistic study revealed that CD63 facilitates LMP1 (EBV‐encoded oncoprotein) exosomal trafficking and enhances noncanonical NF‐κB signaling.^91^ Another interesting newly identified, exosome‐derived miRNA is BART (BamHI fragment A rightward transcript). BART miRNA was recently shown to induce gene expression of IL‐10, TNF‐α, and Arginase‐1 in macrophages possibly contributing to EBV+ lymphoma development in a humanized mouse model,^92^ but it remains to be explored whether BART miRNA plays a role in cHL in humans. Collectively, these studies show that exosomes play a vital role in alternate cell–cell communication especially via CD30 and NF‐kB signaling between malignant H‐RS cells and distant cells that facilitate HL progression and dissemination.

## 3 CONCLUSION

The cHL microenvironment is peculiar with the overall presentation of a suppressed T‐cell immune response. Several cytokines/chemokines, with the majority of them Th2 type, interplay and relay messages between H‐RS cells and infiltrating cells. EBV modulates the tumor milieu of HL and plays a strategic role in immune escape by enrichment of the TME with *T*_*reg*_ cells and associated immunosuppressive cytokines. Exosomes are emerging as new players of H‐RS cell growth and survival. EBV‐associated exosomes present novel targets for the development of diagnostic biomarkers and anticancer therapy. Development of cell line/animal models for pediatric HL would provide a better understanding of the immune cell response against H‐RS cells and the role of circulating T‐ and B‐cells in the pathogenesis of HL leading to focused therapeutic strategies and preventing long‐term side effects.

## AUTHORS' CONTRIBUTIONS

All authors had full access to the data in the study and take responsibility for the integrity of the data and the accuracy of the data analysis. *Conceptualization*, P.N.; *Writing‐Review & Editing*, P.N., D.D.‐M., N.L.

## CONFLICT OF INTEREST

The authors declare no potential conflict of interest.

## ETHICAL STATEMENT

Not applicable.

## Data Availability

Data sharing is not applicable to this article as no new data were created or analyzed in this study.
